# A novel nomogram for predicting mortality risk in young and middle-aged patients undergoing maintenance hemodialysis: a retrospective study

**DOI:** 10.3389/fmed.2024.1508485

**Published:** 2025-01-07

**Authors:** Lei Sun, Yue Zhang, Xinliang Zuo, Yongmei Liu

**Affiliations:** ^1^Chaohu Clinical Medical College of Anhui Medical University, Hefei, China; ^2^Department of Nephrology, Chaohu Hospital of Anhui Medical University, Hefei, China

**Keywords:** young and middle-aged, hemodialysis, end-stage renal disease, risk factors, mortality, prediction model

## Abstract

**Objectives:**

The annual growth in the population of maintenance hemodialysis (MHD) patients is accompanied by a trend towards younger age groups among new cases. Despite the escalating mortality risk observed in MHD patients, there remains a dearth of research focused on young and middle-aged individuals in this cohort, leading to a deficiency in specialized predictive instruments for this demographic. This research seeks to explore the critical determinants impacting mortality risk in young and middle-aged MHD patients and to construct a prediction model accordingly.

**Methods:**

This study involved 127 young and middle-aged patients undergoing MHD in the Blood Purification Center of Chaohu Hospital of Anhui Medical University from January 2019 to January 2022. The follow-up period for each patient ended either at the time of death or on January 31, 2024. Participants were monitored to determine their survival status and categorized into two groups: those who survived (98 patients) and those who deceased (29 patients). Clinical data were gathered for analysis. Logistic regression was utilized to pinpoint independent risk factors for mortality among these patients. Subsequently, a nomogram was established to predict mortality risk. The efficacy of this model was assessed through the area under the receiver operating characteristic curve (AUC-ROC), alongside a calibration curve and the Hosmer–Lemeshow test to examine its fit. Additionally, decision curve analysis (DCA) was conducted to ascertain the clinical relevance of the predictive model.

**Results:**

The study incorporated 127 young and middle-aged patients undergoing MHD, with a mortality rate recorded at 22.83% (29 cases). A logistic regression analysis revealed that age, hemoglobin (HB), serum magnesium (Mg), neutrophil-to-lymphocyte ratio (NLR), and platelet-to-albumin ratio (PAR) were significant independent predictors of mortality among these patients. Utilizing these variables, a nomogram was developed to predict mortality risk, achieving an AUC of 0.899 (95% CI: 0.833–0.966). The model exhibited a specificity of 83.67% and a sensitivity of 82.76%, demonstrating substantial discriminative ability. The model’s robustness was confirmed through internal validation with 1,000 bootstrap samples, yielding an AUC of 0.894 (95% CI: 0.806–0.949). The calibration curve closely aligned with the ideal curve, and the Hosmer–Lemeshow goodness-of-fit test yielded a *χ*^2^ value of 6.312 with a *p*-value of 0.612, verifying the model’s high calibration accuracy. Additionally, the DCA indicated that the model provides a net benefit across a wide range of decision thresholds from 0 to 0.99, underscoring its clinical utility.

**Conclusion:**

The nomogram developed from variables including age, HB levels, serum Mg, NLR, and PAR exhibits high levels of discrimination and calibration. This model effectively predicts mortality risk among young and middle-aged patients undergoing MHD, proving its clinical relevance.

## Introduction

Globally, chronic kidney disease (CKD) is witnessing a consistent rise in both incidence and mortality rates, highlighting its status as a significant public health challenge that demands immediate attention (1). As the disease progresses, kidney function deteriorates, leading to approximately 2% of those with CKD developing end-stage kidney disease (ESKD) (2, 3). At this critical point, sustaining life necessitates renal replacement therapy (RRT) (4). The primary treatments available—hemodialysis (HD), peritoneal dialysis (PD), and kidney transplantation—are designed to enhance the quality of life for affected patients (5).

HD has advanced significantly as a treatment for ESKD, and it is now the predominant RRT employed across various countries and regions (6). The 2021 Annual Report on Blood Purification notes that in China alone, around 749,000 patients with ESKD are receiving maintenance hemodialysis (MHD) (7). Projections suggest that by 2025, this figure will reach 870,000 (8). The growing incidence of new MHD cases underscores a shift towards younger age cohorts, with approximately 80% of patients falling within the young and middle-aged adult categories, averaging 55 years old-a demographic that is on average 10 years younger than patients in the United States and Japan (9). Recent improvements in blood purification technologies have markedly enhanced the survival rates of patients on MHD (10). Nevertheless, the mortality rate among MHD patients, as reported by the Chinese Kidney Disease Report, is alarmingly high at 12.5% (9), representing a 6.5 to 7.9-fold increase compared to the general population (4, 11, 12). Similarly, in the United States, despite superior medical technologies, the mortality rate for MHD patients stands at about 15.9% annually (13). Additionally, the Global Burden of Disease Study from 2017 indicates that over the past two decades, ESKD has emerged as one of the top three fastest-growing causes of mortality worldwide (14), with projections suggesting it will become the fifth most prevalent cause of death by 2040 (15).

In recent times, the nomogram has gained prominence as a clear, easy-to-use predictive instrument extensively employed in clinical settings. It operates by rendering multivariate regression analysis into a visual format, which helps quantify and assess the risk factors and likelihood of clinical event occurrences through cumulative scoring. This visual representation of statistical predictive models is instrumental in enabling clinicians to quickly identify patients at high risk and supports the creation of targeted interventions (1). Despite the development of various clinical risk prediction models aimed at forecasting mortality in MHD patients, there is a notable lack of such predictive tools for younger and middle-aged cohorts. This study is focused on creating a nomogram model and an associated online calculator specifically designed to evaluate the mortality risk among young and middle-aged MHD patients. Utilizing this model allows for a more precise assessment of all-cause mortality risk within this demographic, facilitating the development of early intervention strategies that are customized to their specific needs, thereby enhancing life quality and reducing mortality rates effectively.

## Materials and methods

### Patient selection

A retrospective study was performed on 198 individuals receiving MHD at the Blood Purification Center in Chaohu Hospital of Anhui Medical University over a period from January 2019 to January 2022. The criteria for inclusion involved: (1) age between 18 and 59 years (according to the World Health Organization’s age classification criteria for young and middle-aged individuals) (16); (2) a diagnosis of ESKD with a stable regimen of HD maintained for at least 3 months, occurring thrice weekly; (3) availability of comprehensive clinical records; (4) demonstrated compliance and provision of signed informed consent. The exclusion criteria encompassed: (1) coexisting malignant tumors; (2) severe concurrent infectious diseases; (3) any prior kidney transplantation or peritoneal dialysis; (4) recent acute cardiovascular incidents like acute coronary syndrome or myocardial infarction; (5) any cerebrovascular incidents such as cerebral infarction or hemorrhage occurring within 1 month prior to the study; (6) mental or consciousness disorders impeding cooperation in clinical processes. From the initial cohort, 127 MHD patients qualified for the study, with 33 aged between 18 and 44 (youth), and 94 between 45 and 59 (middle-aged adult). The study received approval from the Ethics Committee of Chaohu Hospital of Anhui Medical University and was aligned with the ethical standards of the Helsinki Declaration.

### Data collection

Patient clinical data were gathered using the hospital’s electronic medical record system, with inclusion criteria shaped by prior research. Collected demographic information encompassed age, gender, etiology of the primary disease, and prevalent comorbid conditions including hypertension, diabetes mellitus (DM), cardiovascular and cerebrovascular diseases (CCD), as well as body mass index (BMI). In the early morning following an overnight fasting period, blood samples were obtained from patients prior before the commencement of regular HD treatment later that day. These samples were then subjected to analysis for various parameters, such as neutrophil count (N), lymphocyte count (L), hemoglobin (HB), red cell distribution width (RDW), platelet count (PLT), pre-albumin (PA), serum albumin (ALB), serum globulin (GLB), alkaline phosphatase (ALP), blood urea nitrogen (BUN), serum creatinine (SCr), cystatin C (CysC), serum uric acid (SUA), total cholesterol (TC), triglycerides (TG), high-density lipoprotein (HDL-C), low-density lipoprotein (LDL-C), serum potassium (K), serum calcium (Ca), serum magnesium (Mg), serum phosphorus (P), homocysteine (Hcy), hypersensitive C-reactive protein (hs-CRP), and intact parathyroid hormone (iPTH). Additionally, dialysis-related parameters included age at initiation of dialysis, dialysis vintage, dialysis shift (morning, afternoon, evening), type of vascular access (autologous arteriovenous fistula, long-term catheter), ultrafiltration volume, and the urea clearance index (Kt/V). Key ratios calculated were the albumin-to-globulin ratio (A/G = ALB/GLB), neutrophil-to-lymphocyte ratio (NLR = N/L), and platelet-to-albumin ratio (PAR = PLT/ALB).

### Follow-up and outcome

The follow-up began upon the completion of laboratory sample collection and was carried out through outpatient dialysis sessions as well as reviews of inpatient medical records. This process continued until January 31, 2024. The principal outcome of interest was patient mortality, which marked the termination of the follow-up phase. During this interval, the survival and mortality statuses of young and middle-aged MHD patients were documented, and individuals were categorized accordingly into either a survival or mortality group.

### Statistical analysis

Quantitative data following a normal distribution are detailed as mean ± standard deviation (SD), whereas data not normally distributed are shown using the median (interquartile) [*M* (*Q*1–*Q*3)]. For group comparisons, the *t*-test or Mann–Whitney *U* test is utilized based on the data’s distribution. Frequencies are reported as *n* (%) and analyzed across groups via the Chi-square test or Fisher’s exact test as appropriate. To identify predictors of long-term outcomes in young and middle-aged MHD patients, a univariate logistic regression analysis was conducted. Subsequently, covariates with a *p*-value <0.05 in the univariate analysis were included in a multivariate logistic regression analysis (using backward stepwise approach) to independent predictors, leading to the creation of a nomogram to estimate mortality risk within this demographic. The efficacy of the model was assessed by the receiver operating characteristic curve (ROC), measuring the area under the curve (AUC) to judge its discriminative ability. Internal consistency was verified through 1,000 bootstrap samples, and model accuracy was evaluated with a calibration curve alongside the Hosmer–Lemeshow test for goodness-of-fit. Decision curve analysis (DCA) was performed to ascertain the model’s clinical utility. Data were analyzed using R software and EmpowerStats,^1^ with a significance threshold established at *p* < 0.05.

## Results

### Baseline characteristics

After establishing inclusion and exclusion criteria, we assembled a cohort of 127 young and middle-aged MHD patients for this investigation, comprising 78 males and 49 females, with ages range from 28 to 59 years and a median age of 51 years. During the 2-year observation period, 29 individuals passed away, representing 22.83% of the total cohort. Comparative analysis unveiled that the mortality group displayed notably higher levels in parameters such as age, comorbidities including DM and CCD, TG, hs-CRP, age at initiation of dialysis, RDW, NLR, and PAR, in contrast to the survival group. Conversely, markers such as A/G, SCr, K, Mg, Ca, Hcy, and HB showcased significantly lower values in the mortality group (*p* < 0.05). However, no noteworthy disparities emerged between the two groups concerning gender distribution, primary disease etiology, concurrent hypertension, BMI, PA, ALB, ALP, BUN, CysC, SUA, TC, HDL-C, LDL-C, P, iPTH, dialysis vintage, dialysis shift, vascular access type, or Kt/V adequacy (*p* > 0.05). Detailed findings are outlined in Table 1.

**Table 1:** Comparison of basic data between the survival group and the deceased group.

Variables	Total	Survival group	Deceased group	*t*/*Z*/*χ*^2^	*p*-value
	(*n* = 127)	(*n* = 98)	(*n* = 29)		
Age [*M* (*Q*_1_–*Q*_3_), years]	51.000 (44.000–55.000)	50.000 (44.000–54.000)	53.000 (49.000–55.000)	2.995	0.004
Gender [*n* (%)]				0.324	0.569
Male	78 (61.420%)	62 (63.265%)	16 (55.172%)		
Female	49 (38.580%)	36 (36.735%)	13 (44.828%)		
Primary disease etiology [*n* (%)]				9.474	0.092
Diabetic nephropathy	17 (13.390%)	9 (9.184%)	8 (27.586%)		
Chronic nephritic syndrome	82 (64.570%)	68 (69.388%)	14 (48.276%)		
Polycystic kidney disease	2 (1.570%)	1 (1.020%)	1 (3.448%)		
Hypertensive nephropathy	2 (1.570%)	2 (2.041%)	0 (0.000%)		
Obstructive nephropathy	2 (1.570%)	2 (2.041%)	0 (0.000%)		
Others	22 (17.320%)	16 (16.327%)	6 (20.690%)		
Hypertension [*n* (%)]				1.868	0.172
Yes	95 (74.800%)	70 (71.429%)	25 (86.207%)		
No	32 (25.200%)	28 (28.571%)	4 (13.793%)		
DM [*n* (%)]				6.083	0.014
Yes	19 (14.960%)	10 (10.204%)	9 (31.034%)		
No	108 (85.040%)	88 (89.796%)	20 (68.966%)		
CCD [*n* (%)]				11.049	<0.001
Yes	42 (11.810%)	6 (6.122%)	9 (31.034%)		
No	156 (88.190%)	92 (93.878%)	20 (68.966%)		
BMI [*n* (%)]				0.676	0.713
<18 kg/m^2^	23 (18.110%)	18 (18.367%)	5 (17.241%)		
18–24 kg/m^2^	80 (62.990%)	63 (64.286%)	17 (58.621%)		
≥24 kg/m^2^	24 (18.900%)	17 (17.347%)	7 (24.138%)		
PA [*n* (%)]				0.018	0.893
<400 mg/L	49 (38.580%)	37 (37.755%)	12 (41.379%)		
≥400 mg/L	78 (61.420%)	61 (62.245%)	17 (58.621%)		
ALB [*n* (%)]				0.015	0.903
<40 g/L	56 (44.090%)	44 (44.898%)	12 (41.379%)		
≥40 g/L	71 (55.910%)	54 (55.102%)	17 (58.621%)		
A/G (mean ± SD)	1.253 ± 0.199	1.283 ± 0.195	1.151 ± 0.182	3.235	0.001
ALP [*n* (%)]				1.308	0.253
<100 U/L	60 (47.240%)	49 (50.000%)	11 (37.931%)		
≥100 U/L	67 (52.760%)	49 (50.000%)	18 (62.069%)		
BUN (mean ± SD, mmol/L)	25.281 ± 6.742	25.867 ± 6.928	23.300 ± 5.746	1.818	0.071
sCr (mean ± SD, μmol/L)	1034.803 ± 249.739	1060.163 ± 252.195	949.103 ± 224.652	2.133	0.035
CysC [*M* (*Q*_1_–*Q*_3_), mg/L]	7.820 (7.325–8.855)	7.825 (7.348–8.732)	7.760 (7.310–9.220)	0.822	0.415
SUA (mean ± SD, μmol/L)	472.307 ± 97.81	476.459 ± 97.029	458.276 ± 100.852	0.879	0.381
TC [*M* (*Q*_1_–*Q*_3_), mmol/L]	4.160 (3.645–4.730)	4.150 (3.555–4.635)	4.190 (3.690–5.120)	0.832	0.411
TG [*M* (*Q*_1_–*Q*_3_), mmol/L]	1.920 (1.300–2.500)	1.835 (1.260–2.385)	2.070 (1.680–3.270)	1.807	0.079
HDL-C [*M* (*Q*_1_–*Q*_3_), mmol/L]	0.960 (0.820–1.165)	0.945 (0.820–1.167)	0.970 (0.840–1.160)	0.199	0.843
LDL-C [*M* (*Q*_1_–*Q*_3_), mmol/L]	2.060 (1.630–2.465)	2.065 (1.672–2.453)	2.040 (1.500–2.470)	0.178	0.860
K [*M* (*Q*_1_–*Q*_3_), mmol/L]	5.000 (4.500–5.700)	5.050 (4.600–5.700)	4.800 (4.140–5.300)	2.450	0.017
Mg [*M* (*Q*_1_–*Q*_3_), mmol/L]	1.090 (1.010–1.190)	1.120 (1.013–1.208)	1.030 (0.970–1.140)	2.450	0.018
Ca [*M* (*Q*_1_–*Q*_3_), mmol/L]	2.360 (2.200–2.505)	2.385 (2.290–2.507)	2.200 (2.120–2.420)	1.787	0.082
P (mean ± SD, mmol/L)	1.980 ± 0.576	2.001 ± 0.568	1.912 ± 0.608	0.729	0.467
Hcy [*M* (*Q*_1_–*Q*_3_), μmol/L]	33.700 (27.250–48.050)	36.000 (27.350–66.100)	29.700 (21.500–41.100)	2.774	0.007
hs-CRP [*M* (*Q*_1_–*Q*_3_), mg/L]	2.680 (1.185–5.735)	2.105 (1.025–4.270)	5.830 (2.440–10.150)	3.474	0.002
iPTH [*M* (*Q*_1_–*Q*_3_), ng/L]	372.000 (167.200–694.350)	354.400 (159.875–674.700)	424.500 (172.200–712.900)	0.225	0.823
Dialysis vintage [*M* (*Q*_1_–*Q*_3_), month]	60.000 (28.500–93.500)	60.000 (29.000–93.750)	59.000 (27.000–87.000)	0.183	0.857
Age at initiation of dialysis [*M* (*Q*_1_–*Q*_3_), years]	44.000 (36.500–48.000)	43.000 (36.000–47.750)	47.000 (43.000–48.000)	2.607	0.011
Dialysis shift [*n* (%)]				2.125	0.346
Morning shift	38 (29.920%)	30 (30.612%)	8 (27.586%)		
Afternoon shift	50 (39.370%)	41 (41.837%)	9 (31.034%)		
Evening shift	39 (30.710%)	27 (27.551%)	12 (41.379%)		
Vascular access [*n* (%)]				0.056	0.813
Autogenous arteriovenous fistula	109 (85.830%)	85 (86.735%)	24 (82.759%)		
Long-term catheter	18 (14.170%)	13 (13.265%)	5 (17.241%)		
Ultrafiltration volume (mean ± SD, L)	2.978 ± 0.959	2.948 ± 0.913	3.079 ± 1.112	0.646	0.519
Kt/V (mean ± SD)	1.399 ± 0.347	1.413 ± 0.319	1.353 ± 0.434	0.810	0.419
HB (mean ± SD, g/L)	116.315 ± 19.286	118.633 ± 18.531	108.483 ± 20.046	2.543	0.012
RDW [*M* (*Q*_1_–*Q*_3_), %]	13.700 (13.150–14.500)	13.600 (13.100–14.400)	14.300 (13.200–15.100)	2.235	0.032
NLR [*M* (*Q*_1_–*Q*_3_)]	3.232 (2.548–4.224)	3.021 (2.414–3.680)	4.910 (3.846–5.884)	4.452	<0.001
PAR (mean ± SD)	4.392 ± 1.371	4.200 ± 1.321	5.044 ± 1.358	3.007	0.003

DM, diabetes mellitus; CCD, cardiovascular and cerebrovascular disease; BMI, body mass index; PA, pre-albumin; ALB, serum albumin; A/G, albumin-to-globulin ratio; ALP, alkaline phosphatase; BUN, blood urea nitrogen; SCr, serum creatinine; CysC, cystatin C; SUA, serum uric acid; TC, total cholesterol; TG, triglyceride; HDL-C, high-density lipoprotein; LDL-C, low-density lipoprotein; K, serum potassium; Mg, serum magnesium; Ca, serum calcium; P, serum phosphorus; Hcy, homocysteine; hs-CRP, hypersensitive C-reactive protein; iPTH, intact parathyroid hormone; HB, hemoglobin; RDW, red cell distribution width; NLR, neutrophil-to-lymphocyte ratio; PAR, platelet-to-albumin ratio. Mean ± standard deviation (SD).

### Selection of variables

**Figure 1:**
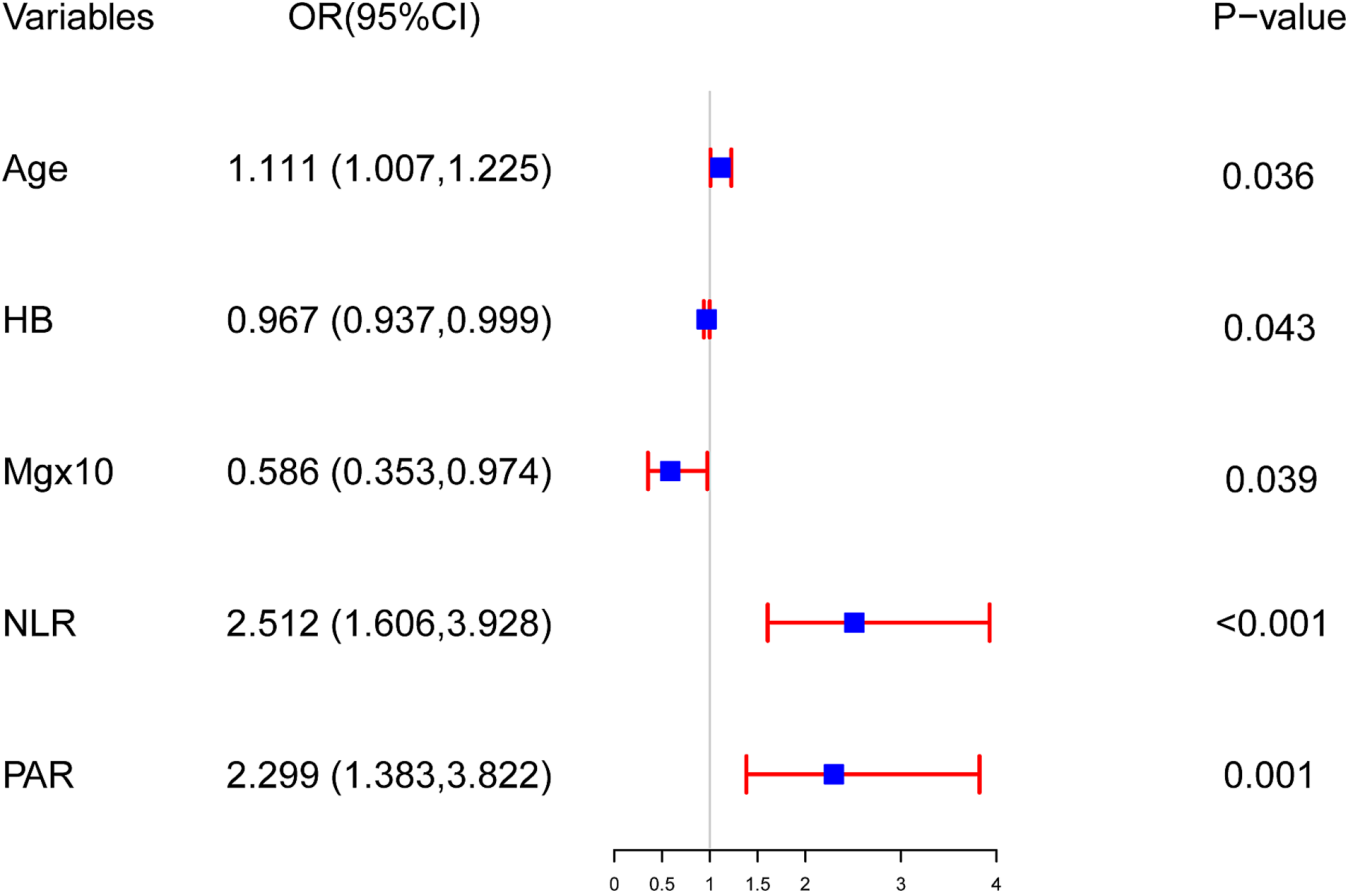
The outcomes of multivariable logistic regression analysis evaluating mortality risk in young and middle-aged patients undergoing maintenance hemodialysis. Logistic regression model: −4.337 + 0.105 × Age − 0.033 × HB − 5.340 × Mg + 0.921 × NLR + 0.832 × PAR.

Based on univariate logistic regression analysis where mortality risk served as the dependent variable and various risk factors as independent variables, those (age, DM, CCD, HB, RDW, A/G, SCr, TG, K, Mg, hs-CRP, age at initiation of dialysis, NLR, and PAR) with *p*-values <0.05 underwent subsequent multivariate logistic regression analysis (detailed in Table 2). The results indicate that advanced age, decreased levels of HB and Mg, along with elevated NLR and PAR contribute to mortality among young and middle-aged patients undergoing MHD (*p* < 0.05) (detailed in Figure 1).

**Table 2:** Univariate logistic regression analysis of risk factors for mortality in young and middle-aged patients undergoing maintenance hemodialysis.

Variables	OR^a^	95% CI^a^	*p*-value
Age (years)	1.080	1.009–1.156	0.026
DM	3.960	1.424–11.016	0.008
CCD	6.900	2.205–21.587	0.001
HB (g/L)	0.972	0.950–0.994	0.015
RDW (%)	1.546	1.111–2.151	0.010
A/G	0.026	0.002–0.278	0.003
SCr (μmol/L)	0.998	0.996–1.000	0.038
TG (mmol/L)	1.439	1.034–2.003	0.031
K (mmol/L)	0.544	0.304–0.972	0.040
Mg (mmol/L)	0.012	0.000–0.518	0.021
hs-CRP (mg/L)	1.242	1.108–1.392	<0.001
Age at the start of dialysis (years)	1.057	1.002–1.116	0.043
NLR	2.199	1.550–3.120	<0.001
PAR	1.582	1.150–2.177	0.005

a OR, odds ratio; CI, confidence interval.

### Nomogram for predicting mortality

**Figure 2:**
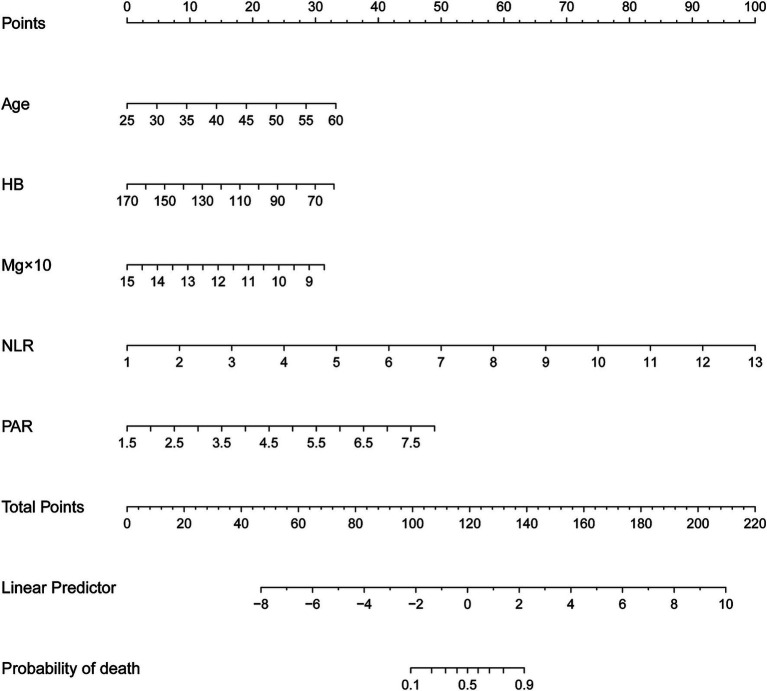
Nomogram for predicting the risk of all-cause mortality among young and middle-aged patients undergoing maintenance hemodialysis. First, score each individual’s predictor value using the top scale. Second, sum up all the scores and identify the corresponding total score on the scale. Finally, the risk of death for the given young and middle-aged patient undergoing maintenance hemodialysis corresponds to the corresponding risk on the lowest scale. HB, hemoglobin; Mg, serum magnesium; NLR, neutrophil-to-lymphocyte ratio; PAR, platelet-to-albumin ratio.

A nomogram model was created from the findings of the logistic regression analyses to estimate the mortality risk among patients undergoing MHD who are in their youth and middle age. This model quantifies the influence of each identified risk factor by assigning scores. In this scoring system, the NLR is the most heavily weighted factor, receiving a maximum of 100 points for a value of 13. The PAR follows with 49 points at a value of 8. A drop in HB to 60 g/L results in a 33-point assignment, the same score is given when a patient’s age reaches 60 years, and a decrease in serum Mg to 0.85 mmol/L also yields 31 points (depicted in Figure 2).

### Validation of the nomogram

**Figure 3:**
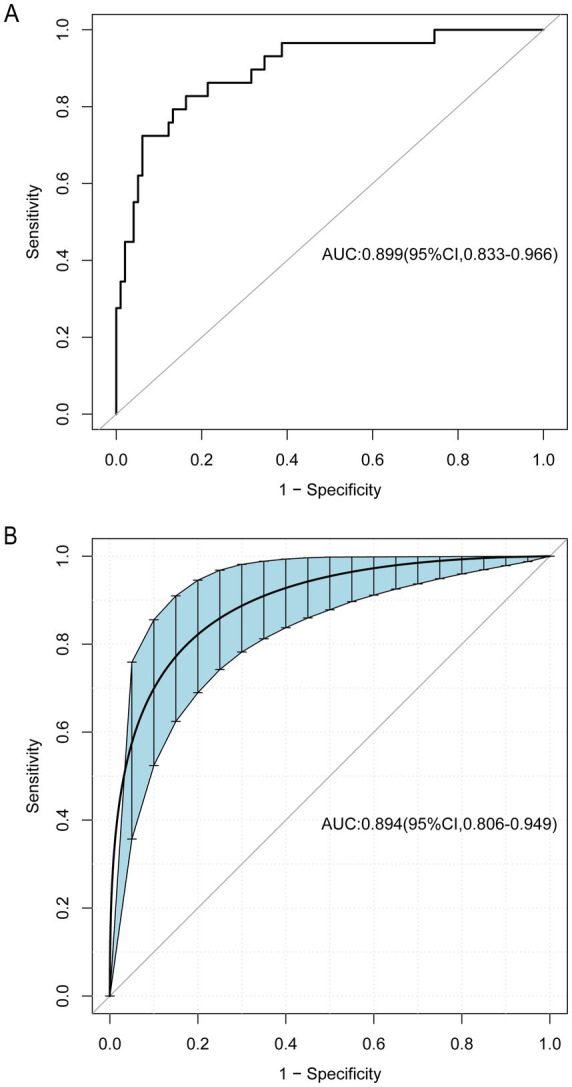
The receiver operating characteristic (ROC) curve for the predictive model, as well as for the internal validation model. The area under the curve (AUC) **(A)** indicates the discriminative capability of the model, while AUC **(B)** pertains specifically to the internal validation model. The shaded blue section depicts the 95% confidence interval. CI, confidence interval.

**Figure 4:**
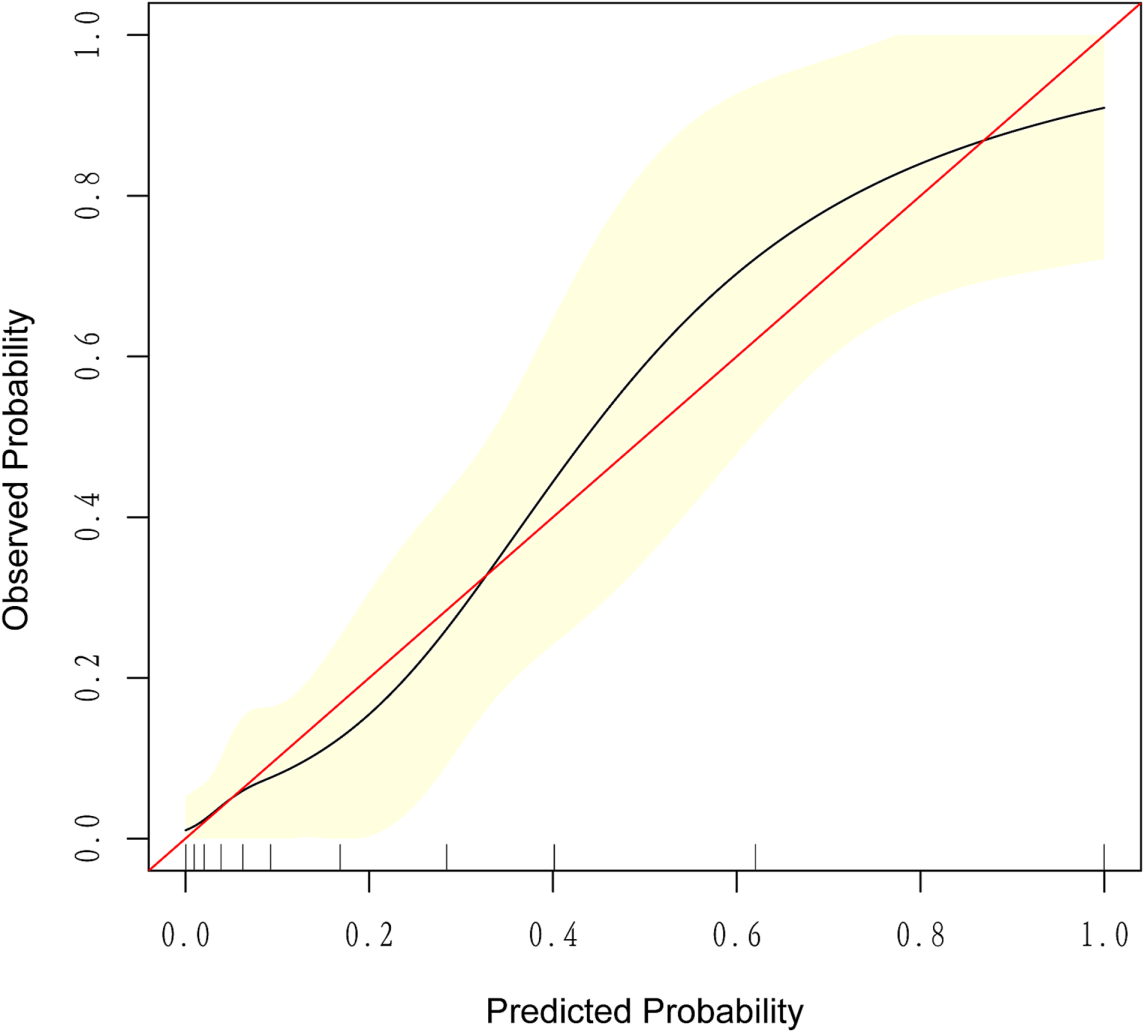
The calibration curve for predicting the probability of all-cause mortality among young and middle-aged patients undergoing maintenance hemodialysis. It demonstrates a good fit between the predicted risk of death and observed outcomes among young and middle-aged patients undergoing maintenance hemodialysis. The solid red line represents an ideal predictive model, while the solid black line indicates the actual performance of the predictive model. The yellow shading represents a 95% confidence interval.

**Figure 5:**
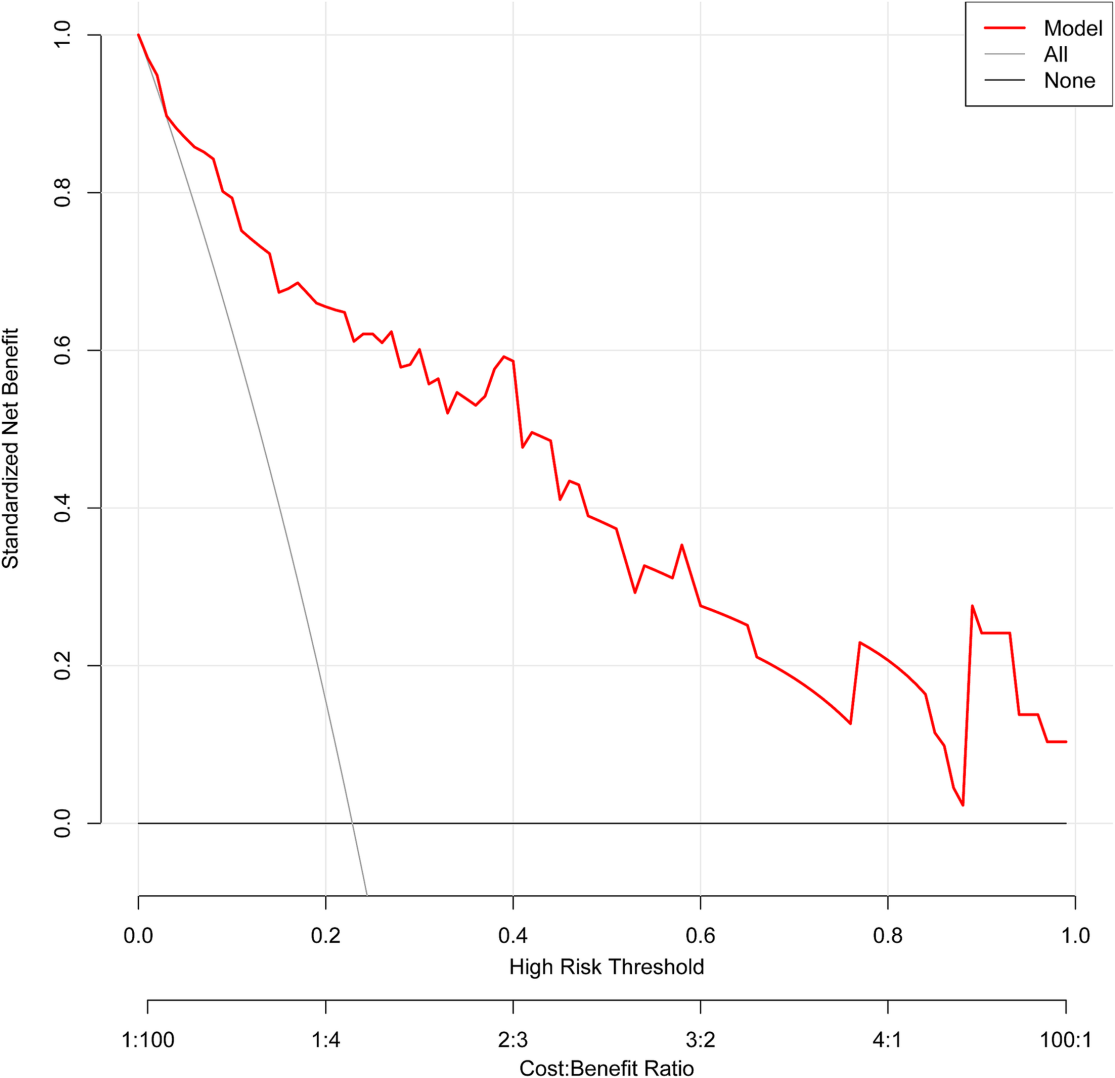
The decision curve analysis of the predictive model. The net benefit is generated based on the high-risk threshold. The solid red line represents the predicted estimates. The decision curve suggests that when the threshold probability falls between 0 and 99%, using this model for decision-making is more beneficial to patient outcomes compared to either the “treat-all” or “treat-none” strategies.

The ROC curve analysis demonstrated that the AUC for the mortality risk prediction model in young and middle-aged MHD patients stands at 0.899, with a 95% confidence interval (CI) ranging from 0.833 to 0.966 (as shown in Figure 3A). This model exhibits a specificity of 83.67% and a sensitivity of 82.76%. The AUC for the internal validation of this model is recorded at 0.894 with a 95% CI between 0.806 and 0.949 (illustrated in Figure 3B). The alignment between predicted and observed mortality risks in this demographic is confirmed by the calibration curve (as depicted in Figure 4). The Hosmer–Lemeshow test for goodness of fit indicates a high model accuracy with a *χ*^2^ value of 6.312 and a *p*-value of 0.612. The DCA validates the clinical utility of the nomogram in predicting mortality risk among young and middle-aged MHD patients (as depicted in Figure 5). Setting the threshold probability range of the model between 0 and 0.99 reveals a net benefit above zero, substantiating the model’s efficacy.

## Discussion

This study employed both univariate and multivariate logistic regression analyses to identify independent mortality risk factors in young and middle-aged MHD patients. These factors included age, HB, serum Mg, NLR, and PAR. Utilizing these variables, a novel, simple, and practical nomogram was developed to assess mortality risk. The model demonstrated excellent discriminatory capabilities, with an AUC of 0.899, along with a sensitivity of 82.76% and specificity of 83.67%. Internal validation through 1,000 bootstrap resampling resulted in an AUC of 0.894. The calibration analysis indicates a strong agreement between the predicted probabilities and actual outcomes, with the calibration curve closely matching the ideal diagonal line. This suggests that the model provides reliable risk predictions across the range of probabilities assessed, enhancing its potential for clinical use. However, small discrepancies in certain risk categories highlight the importance of conducting external validation in diverse populations. Furthermore, the DCA demonstrates the model’s clinical relevance, showing a higher net benefit across a probability threshold range of 0 to 0.99 compared to traditional approaches like “treat all” or “treat none.” This suggests that the model effectively balances the advantages and risks of interventions, particularly in guiding targeted care for patients at varying risk levels. Still, further investigation of the model’s external validation in populations from varied geographical regions and ethnic groups is needed to confirm its generalizability and stability.

Extensive research has established that HB levels critically indicate the risk of all-cause mortality among MHD patients, highlighting their importance (1, 17, 18). This study identifies reduced HB as an independent risk factor for mortality in young and middle-aged MHD patients [odds ratio (OR) = 0.967, *p* = 0.043]. This discovery not only corroborates findings from prior studies but also emphasizes the critical role of HB levels in this patient demographic. In CKD, the development of anemia involves various complex factors. Notably, anemia is linked to a relative shortage of erythropoietin as well as to the sufficiency of dialysis treatment (1). Low erythropoietin levels hinder the production of red blood cells, thereby contributing to anemia. Concurrently, the adequacy of dialysis treatment significantly influences anemia’s severity; insufficient dialysis can lead to an accumulation of toxins and metabolic waste, aggravating the symptoms of anemia.

Age markedly influences the prognosis of patients undergoing MHD (6, 19, 20). Notably, there are substantial variations among different age groups regarding disease progression, treatment outcomes, and quality of life. This investigation establishes age as an independent predictor of mortality in young and middle-aged MHD patients (OR = 1.111, *p* = 0.036). This finding aligns closely with the research conducted by Chang et al. (21), Sant’Ana et al. (22), and Ouyang et al. (4). Older patients receiving hemodialysis typically experience increased complications, diminished immune capabilities, cognitive deterioration, and reduced quality of life, all of which contribute to higher mortality rates. This situation is particularly acute for elderly MHD patients who reside alone and lack adequate support during and after treatment, heightening their mortality risk (4, 23). Thus, enhancing the care and attention provided to these individuals is essential to improve their treatment outcomes and life quality.

Serum Mg levels play a pivotal role in the survival of patients undergoing MHD. A decade-long study by Shimohata et al. (24) on MHD patients with non-diabetic nephropathy categorized participants into groups with high and low serum Mg, based on a critical threshold of 2.5 mg/dL. Findings indicated significantly lower survival rates in the low Mg group compared to their counterparts in the high Mg group. Building on this, Lu et al. (25) examined the link between decreased serum Mg and increased short-term mortality in older MHD patients, confirming a strong correlation. This research underscores that low serum Mg serves as an independent risk factor for mortality in young and middle-aged MHD patients (OR = 0.586, *p* = 0.039), corroborating prior studies. However, findings by Mizuiri et al. (26) suggest a contrasting scenario where low serum Mg (≤2.4 mg/dL) correlated with better three-year survival outcomes concerning all-cause and cardiovascular mortality, although it did not emerge as an independent risk factor for all-cause mortality. Additionally, a Japanese study (27) described a “J”-shaped relationship between serum Mg levels and mortality risk, highlighting significant mortality risk when serum Mg was below 2.7 mg/dL or above 3.1 mg/dL. Our results did not replicate these findings, possibly due to differences in study design, sample selection, or analytic approaches. Extant research posits that serum Mg levels can influence MHD patient survival by impeding vascular calcification (26). Moreover, low serum Mg may be linked to adverse factors like malnutrition and reduced intake, negatively impacting patient outcomes (25). Thus, maintaining optimal serum Mg levels is crucial for enhancing the survival and prognosis of MHD patients.

The NLR is increasingly acknowledged as a critical biomarker for systemic inflammation, demonstrating a significant capability to predict the onset and progression of severe illnesses, including myocardial infarction and cancer, and also indicating potential mortality risks (3, 28, 29). Research conducted by Woziwodzka et al. (30) has established a robust correlation between elevated NLR and increased long-term all-cause mortality in patients at CKD stage 5, notable even when white blood cell counts remain normal. Similarly, findings by Mureșan et al. (31) reveal that NLR values at admission offer a strong predictive measure for 30-day all-cause mortality among ESKD patients who require prolonged RRT exceeding 6 months. Our investigations align with these findings, showing that NLR acts as an independent mortality risk factor in young and middle-aged MHD patients (OR = 2.512, *p* < 0.001). These findings corroborate earlier research, underscoring the critical significance of NLR in the prognosis of CKD patients. Elevated NLR levels may exacerbate the risk of mortality in MHD patients by promoting tumor development and the advancement of cardiovascular diseases, thereby endangering their health and survival (3).

The PAR offers superior stability over individual measurements of PLT and ALB across different physiological and pathological conditions, making it a more precise indicator of inflammation and nutritional status (32). Consequently, PAR has been identified as potentially crucial for prognostic evaluations in patients with CKD and IgA nephropathy (IgAN). Research by Sági et al. (33) in Hungary supports the utility of PAR in forecasting the onset of ESKD and cardiovascular incidents in patients with IgAN. Furthermore, a study by Yang et al. (34) illustrates the significance of PAR in predicting both technique failure and mortality among CKD patients receiving PD. In our analysis, PAR emerged as an independent predictor of mortality among young and middle-aged patients undergoing MHD (OR = 2.299, *p* = 0.001). Beyond its applications in CKD and IgAN, PAR has demonstrated predictive relevance for acute kidney injury post-cardiac surgery in the critically ill (35) and prognostic utility in cases of non-small cell lung cancer (36). This underscores the broad potential of PAR in disease prognostication, particularly highlighting its implications for the prognosis of MHD patients. These findings underscore the necessity for further exploration and confirmation in subsequent studies.

This investigation presents several significant advantages. Primarily, the introduction of a nomogram prediction model designed specifically for evaluating mortality risks in young and middle-aged MHD individuals represents a pioneering tool that facilitates timely interventions aimed at reducing mortality rates among the younger demographic of MHD patients. Additionally, the study establishes PAR as an independent predictor of mortality in PD patients and is the first to apply PAR in assessing all-cause mortality risks in MHD patients, thereby reinforcing the vital role of PAR in the prognostic landscape of CKD patients. Nevertheless, this investigation is constrained by certain limitations. First, it is a single-center, retrospective analysis with a relatively small cohort, which could heighten the susceptibility to type II errors. Despite the development of a robust nomogram model for predicting mortality in young and middle-aged MHD individuals, validated internally through bootstrap techniques, the absence of external validation raises concerns about its applicability across different MHD populations. Future research should focus on expanding the sample size and employing multicenter, prospective methodologies to enhance the reliability and generalizability of the findings. Second, the current analysis was restricted to variables with *p*-values below 0.05 in univariate analyses for logistic regression. This method may neglect some crucial risk factors that did not reach statistical significance individually but could significantly influence mortality outcomes. Thus, extensive further investigations with larger cohorts are essential to assess these factors thoroughly and confirm the model’s accuracy. Third, electrolyte assessments are routinely conducted as part of the regular monitoring for MHD patients in our hospital, with serum Mg concentration being a critical indicator. However, this test is not always included in standard checkups in some hospitals, which may restrict its potential for clinical prediction in those settings. Fourth, this study did not include data related to nutritional status, dietary habits, or medication use during dialysis, which are pertinent to patient outcomes. Future studies are planned to investigate these associations to further refine the understanding of factors influencing mortality in patients of young and middle-aged demographics undergoing MHD.

## Conclusion

This study developed and internally validated a prediction model to assess mortality risk among young and middle-aged MHD patients. The model includes age, HB, serum Mg, NLR, and PAR, all of which demonstrate significant predictive accuracy. These easily accessible factors in clinical practice provide valuable insights for mortality risks evaluation in MHD patients.

## Data Availability

The original contributions presented in the study are included in the article/supplementary material, further inquiries can be directed to the corresponding author.

## Footnotes

^1^ www.empowerstats.com
